# Antioxidant and Antibacterial Activity of Caprylic Acid Vanillyl Ester Produced by Lipase-Mediated Transesterification

**DOI:** 10.4014/jmb.2010.10018

**Published:** 2020-11-10

**Authors:** Jin Ju Kim, Hyung Kwoun Kim

**Affiliations:** Division of Biotechnology, The Catholic University of Korea, Bucheon 14662, Republic of Korea

**Keywords:** Butyric acid vanillyl ester, caprylic acid vanillyl ester, antioxidant, antibacterial activity, lipase

## Abstract

Vanillyl alcohol (VA), which is abundant in Vanilla bean, has strong antioxidant activity. However, the use of VA in the food and cosmetics industries is limited, due to its low solubility in emulsion or organic solvents. Meanwhile, medium chain fatty acids and medium chain monoglycerides have antibacterial activity. We synthesized butyric acid vanillyl ester (BAVE) or caprylic acid vanillyl ester (CAVE) from VA with tributyrin or tricaprylin through transesterification reaction using immobilized lipases. BAVE and CAVE scavenged 2,2-diphenyl-1-picrylhydrazyl radicals in organic solvents. In addition, BAVE and CAVE decreased the production rate of conjugated diene and triene in the menhaden oil-in-water emulsion system. While BAVE showed no antibacterial activity, CAVE showed antibacterial activity against food spoilage bacteria, including *Bacillus coagulans*. In this study, the antibacterial activity of vanillyl ester with medium chain fatty acid was first revealed. Zeta potential measurements confirmed that BAVE and CAVE were inserted into *B. coagulans* membrane. In addition, the propidium iodide uptake assay and fluorescent microscopy showed that CAVE increased *B. coagulans* membrane permeability. Therefore, CAVE is expected to play an important role in the food and cosmetics industries as a bi-functional material with both antioxidant and antibacterial activities.

## Introduction

Antioxidants have been added for a long time to food to preserve food stuff. In the past, butylated hydroxyanisole (BHA) and butylated hydroxytoluene (BHT) were used in the food industry as synthetic antioxidants, but due to potential toxicity, such as liver damage or carcinogenicity, their use is now limited [1]. Therefore, the need for new natural antioxidants has been emerging [2, 3]. Natural antioxidants developed so far include β-carotene, ascorbic acid, and tocopherol. Recently, some phenolic acids/alcohols have also been attracting much attention as natural antioxidants. For example, phenolic acid extracted from kale leaves and seeds showed activity to remove 1,1-diphenyl-2-picrylhydrazyl (DPPH) radicals [4]. However, most phenolic acids/alcohols have low solubility for non-polar solvents, which has limited their use in food. Therefore, it is necessary to use enzyme-based lipophilization to increase hydrophobicity [5]. For example, the antioxidant activity of phenolic lipids synthesized by the esterification of rutin and vanillyl alcohol with the ω-3 polyunsaturated fatty acids extracted from fish byproducts was reported [6].

Another important subject in the food industry is the prevention of food spoilage bacteria [7-11]. *Bacillus subtilis* and *Bacillus coagulans* are Gram-positive bacteria that cause food spoilage. *B. coagulans*, in particular, are known to cause flat-sour spoilage of canned foods [12, 13]. *Alcaligenes faecalis* and *Pseudomonas fluorescens* are known as gram-negative bacteria that decompose food [12]. Therefore, it is necessary to develop efficient antibacterial substances that inhibit the growth of these bacteria. Moreover, as the emergence of antibiotic-resistant bacteria has become a global issue, the development of a new type of natural antibiotics has become important [14-18]. Recently, various antibiotic substitutes have been screened from lipid materials that bacteria do not develop resistance [19]. For example, some fatty acids, such as polyunsaturated fatty acid [20] and hydroxy fatty acid [21], are potential candidates. In addition, medium chain fatty acid and medium chain monoglycerides have been reported to also have antibacterial activity [22, 23].

Capsaicinoid has been known to have various biological activities, such as antibacterial and antioxidant [24, 25]. However, since the use of capsaicinoids in food and beverage industries is limited due to their pungency [26], studies on capsinoids that share many biological activities have been conducted [27]. Capsinoid is phenolic lipid, in which fatty acid is linked to vanillyl alcohol by ester bond [28]. The structural difference between capsinoid and capsaicinoid is in the bond between the fatty acid and the aromatic ring portion: capsaicinoid is the amide bond, and capsinoid is the ester bond [29]. Recently, ricinoleic acid vanillyl ester, a type of capsinoid synthesized by the transesterification of VA and castor oil, was reported to exhibit antibacterial activity against food spoilage bacteria [21].

Lipase catalyzes the hydrolysis of long chain triglycerides in aqueous solution. It also catalyzes esterification, interesterification, and transesterification in organic solvents [30]. Most of these reactions use immobilized lipases, which are advantageous because of enhanced chemical, mechanical, and thermal stability, reusability, and cost reduction [31, 32].

In this study, we synthesized butyric acid vanillyl ester (BAVE) and caprylic acid vanillyl ester (CAVE) by enzymatic transesterification using vanillyl alcohol with tributyrin or tricaprylin. In addition, we evaluated the antioxidant and antibacterial activities of BAVE and CAVE, and investigated the antibacterial mechanism of CAVE, using zeta potential, propidium iodide uptake assay, and fluorescent microscopy.

## Materials and Methods

### Chemicals

Vanillyl alcohol (4-hydroxy-3-methoxybenzyl alcohol, VA), glyceryl trioctanoate (tricaprylin, TCN), glyceryl tributyrate (tributyrin, TBN), 2,2-diphenyl-1-picrylhydrazyl (DPPH), 2,6-di-*tert*-butyl-4-methylphenol (BHT), Menhaden oil (MO), 1-butanol, and bacterial propidium iodide (PI) solution were purchased from Sigma-Aldrich Co. (USA). Acetone and toluene were purchased from Junsei Co. (Japan), while methanol was purchased from Merck Co. (Germany). Methanol and HPLC grade water were purchased from Samchun Chemicals Co.(Korea), and methacrylate-divinylbenzene (MA-DVB) resin was purchased from GenoFocus (Korea). LB broth was purchased from Becton, Dickinson and Co. (USA).

### Enzymes

*Candida antarctica* lipase (CalB) immobilized on acrylic resin was purchased from Sigma-Aldrich Co. (USA). Recombinant *Proteus vulgaris* K80 lipase was expressed in *Escherichia coli* BL21 (DE3) cell, and the K80 lipase in the cell-free extract was immobilized on MA-DVB resin [33].

### Bacterial Strains

*Bacillus coagulans* (KCCM 11715) and *Pseudomonas fluorescens* (KCCM 41443) were purchased from the Korea Culture Center of Microorganisms (KCCM, Korea), while *Alcaligenes faecalis* (KCTC 2678) and *Bacillus subtilis* (KCTC 2189) were purchased from the Korea Collection for Type Cultures (KCTC, Korea).

### Synthesis of BAVE and CAVE Using CalB Lipase

Firstly, to optimize the molar ratio of VA and TBN (or TCN), 30 mM VA, (10–150) mM TBN (or TCN), 30 mg CalB lipase (2.7 U/g of bead), and 100 mg molecular sieve were added to 5 ml acetone. Reactions were conducted at 55°C for 4 h at 210 rpm, and the reaction mixtures were analyzed by HPLC.

Secondly, to optimize the enzyme amount, 30 mM VA, 90 mM TBN (or 60 mM TCN), (2–40) mg CalB lipase, and 100 mg molecular sieve were added to 5 mL acetone. Reactions were conducted at the same conditions as indicated above.

Finally, to optimize the reaction time, 30 mM VA, 90 mM TBN, 10 mg CalB lipase (or 60 mM TCN, 20 mg CalB lipase), and 100 mg molecular sieve were dissolved in 5 mL acetone. Reactions were conducted at the same conditions as mentioned above. The reaction solution samples were taken at the predetermined times of up to 6 h, and analyzed by HPLC.

To measure the BAVE and CAVE amount, reaction mixtures were filtered through a 0.45 μm membrane (NORM-JECT Syringe, nylon filter media, Whatman TM), prior to HPLC analysis.

### Synthesis of BAVE and CAVE Using K80 Lipase

Firstly, to optimize the molar ratio of VA and TBN (or TCN), 30 mM VA, (10–210) mM TBN (or TCN), 0.25 U K80 lipase (10. 7 U/g of bead), and 100 mg molecular sieve were added to 5 ml acetone. Reactions were conducted at 30°C for 4 h at 210 rpm.

Secondly, to optimize the enzyme amount, 30 mM VA, 150 mM TBN (or 180 mM TCN), (0.125–1) U K80 lipase, and 100 mg molecular sieve were added to 5 ml acetone. Reactions were conducted under the same conditions as indicated above.

Finally, to optimize the reaction time, 30 mM VA, 150 mM TBN, 0.75 U K80 lipase (or 180 mM TCN, 0.5 U K80 lipase), and 100 mg molecular sieve were dissolved in 5 ml acetone. Reactions were conducted at the same conditions as indicated above. The reaction solution samples were taken at the predetermined times of up to 6 h, and analyzed by HPLC.

### Analytical Method

Substrates and products in the reaction mixture were monitored using Agilent 1100 series HPLC, and separation of the substrates and products was performed using a Cogent Bidentate C18 column (4.6 mm × 250 mm, 5 μm particle size; microSolv Technology Corp., USA). Elution was performed using a gradient of solvent A (methanol/water, 70/30, v/v) and solvent B (methanol) at a flow rate of 0.9 ml/min. The elution protocol of BAVE was as follows: (0–20) min: 100% A. The elution protocol of CAVE was as follows: (0–5) min: 100% A; (5–10) min: (100–0) % A in B; (10–20) min: 100% B; (20–25) min: (100–0) % B in A; (25–30) min: 100% A. All components in reaction mixtures were detected at 215 nm. Conversion yield (%) and products concentration were calculated based on the decrease of VA. Conversion yield (%) was calculated by the following Eq. (1):

$$\mathrm{Conversion}\mathrm{yield}(\%)=\frac{A_{t0}-A_{t}}{A_{t0}}\times 100$$

where, *A*_to_ is the peak area of VA at 0 h, and *A*_t_ is the peak area of VA at a given time t.

### Purification of Products

Purification of the products was performed by preparative LC, using XBridge C18 column (50 mm × 250 mm, 5 μm particle size; Waters Corp., USA) at a flow rate of 70 ml/min. The elution protocol of BAVE was as follows:(0–20) min: 100% B. The elution protocol of CAVE was as follows: (0–5) min: 100% A; (5–10) min: (100–0) % A in B; (10–30) min: 100% B. The fractions between (3.5–4.5) min for BAVE and (13–14) min for CAVE were collected. Samples collected from the column were concentrated using centrifugal evaporator (EYELA N1000V, Japan), to yield a viscous and yellow oily liquid.

### Structure Analysis of Butyric Acid Vanillyl Ester

The NMR spectra were measured by Avance III 300 MHz (Bruker BioSciences Corp., USA). ^1^H-NMR (CDCl_3_) δ: (0.912 – 0.983) (m, 3H, 1-CH_3_), (1.583 – 1.704) (m, 2H, 2-CH_2_), (2.276 – 2.361) (m, 2H, 3-CH_2_), 3.898 (s,rOCH_3_), (4.123 – 4.330) (m,lyceryl 2CH_2_, impurity; from TBN), 5.028 (s,rCH_2_O), (5.244 – 5.313)(m,lyceryl CH, impurity; from TBN), 5.644 (s,rOH), (6.856 – 6.885) (m, 3H, 3ArH).

### Structure Analysis of Caprylic Acid Vanillyl Esters

The NMR spectrum of CAVE matched well with the previously reported literature [34]. ^1^H-NMR (CDCl_3_) δ: 0.868 (t, *J*=0.69 Hz, 3H, 1-CH_3_), (1.240 – 1.312) (m, 8H, 3-6-(CH_2_)4), (1.577 – 1.650) (m, 2H, 2-CH_2_), (2.302 –2.352) (t, J=7.5 Hz, 2H, 7-CH_2_), 3.900 (s,rOCH_3_), 5.026 (s,rCH_2_O), 5.632 (s,rOH), (6.859 –6.915) (m, 3H, 3ArH).

### Determination of DPPH Radical Scavenging Activity

DPPH radical scavenging activities of VA, TBN, TCN, BAVE, and CAVE were determined according to the previously reported method [20, 21]. The residual DPPH radical percentage was calculated by the following equation:

$$\mathrm{Residual}\mathrm{DPPH}\mathrm{radical}(\%)=\frac{A_{\mathrm{sample}}}{A_{\mathrm{control}}}\times 100$$

where, *A*_sample_ is the absorbance of sample at 517 nm, and *A*_control_ is the absorbance of the negative control at 517 nm.

### Determination of Conjugated Diene and Triene in MO-in-Water Emulsion

The ability of VA, TBN, TCN, BAVE, and CAVE to prevent conjugated diene (CD) and conjugated triene (CT) formation in menhaden oil-in-water emulsions was determined according to the previously reported literature [20, 35]. MO-in-water emulsions containing 1% Tween 20 and 10% MO were prepared by sonication in ice. To get 1 mM concentration of products (or substrates), each compound in acetone was added into a glass vial, and acetone was removed using a centrifugal evaporator, before the addition of MO-in-water emulsions (5 ml). The mixtures were subsequently sonicated for total dispersion of the test compound. All emulsions were kept at 35°C for 5 days in the dark with agitation of 160 rpm. Absorbance was measured at 237 nm for CD, and 270 nm for CT. The results were calculated as mmol of CD or CT per milliliter of emulsion, based on the Beer–Lambert Law.

### Partition Behavior of Products

 MO-in-water emulsion was prepared by mixing 50% MO and 1 mM of each sample with the addition of 1%Tween 20 as an emulsifier. The solution was vortexed for 2 min, and stored at 20°C for 24 h. Then, emulsion (upper) and aqueous (lower) phases were separated, and analyzed using HPLC. The percentages of VA, BAVE, and CAVE partition in emulsion phase were calculated using the following equation:

$$\mathrm{Partition}\mathrm{in}\mathrm{emulsion}\mathrm{phase}(\%)=\frac{A_{E}}{A_{E}+A_{A}}\times 100$$

where, A_E_ is the peak area of each compound in emulsion phase, and A_A_ is the peak area of each compound in aqueous phase.

### Determination of Minimum Inhibitory Concentration

Four strains of food spoilage bacteria, *i.e.*, *A. faecalis*, *B. coagulans*, *B. subtilis*, and *P. fluorescens*, were used to determine the minimum inhibitory concentration (MIC) of products. The antibacterial activity was tested according to the previously described method with slight modification [14]. The bacteria were grown for 16 h at 30°C (*B. subtilis* and *P. fluorescens*) or 37°C (*A. faecalis* and *B. coagulans*) in LB broth medium, from which an inoculum was taken, and adjusted to an OD_640nm_ of 0.2 (1 × 10^8^ CFU/ml). It was then diluted 500 times, and added into the cap tubes. Serial dilutions of test compound in acetone were added to the cap tube. The final concentration of test compound was (1.75–1,000) μM, and the final acetone percentage was 2.5% (*A. faecalis*) or 5% (*B. coagulans*, *B. subtilis* and *P. fluorescens*). Subsequently, each bacterium was incubated at (30 or 37) °C for 24 h. The turbidity was then determined at 640 nm. The inoculum with (2.5 or 5) % acetone was also incubated as a control. The MIC was defined as the concentration that, compared with control, inhibits 90% growth of bacteria.

### Determination of Bacterial Cell Membrane Zeta-Potential

*B. coagulans* cells cultured for 16 h were washed twice with PBS. Washed cells were adjusted to an OD_640nm_ of 0.2, and diluted 500 times. BAVE and CAVE (2 mM each) were treated, and cell suspensions were incubated for 4 h at 37°C. After incubation, bacterial cells were centrifuged at 12,000 rpm for 5 min at 4°C. Cell pellets were suspended with PBS. Bacterial cells treated with 5% acetone (or nothing) were also used as controls. Cell suspensions were loaded into a folded capillary zeta cell of a particle electrophoresis instrument (Zetasizer Nano ZA, Malvern Instruments Ltd, UK).

### Propidium Iodide (PI) Uptake Assay

The PI uptake of *B. coagulans* treated with BAVE or CAVE was measured according to the previously reported method with slight modification [36]. *B. coagulans* cells cultured for 16 h were washed twice with PBS. Washed cells were adjusted to an OD_640nm_ of 0.2, BAVE or CAVE (1 mM each) was treated, and cell suspensions were incubated for 4 h at 37°C. After incubation for 4 h at 37°C, bacterial cells were centrifuged at 12,000 rpm for 5 min at 4°C. Cell pellets were suspended with PBS. PI (3 μM) was treated to the cell suspension. After incubation for 20 min in the dark, cells were centrifuged at 12,000 rpm for 5 min at 4°C. The supernatant was discarded, and cell pellet was suspended with PBS. The fluorescence of suspensions was measured by fluorescence spectrophotometry (synergy MX, BioTek, USA), at an excitation of 544 nm and emission of 620 nm.

### PI Staining and Fluorescent Microscopy

*B. coagulans* cells were treated with BAVE and CAVE as PI uptake assay. The PI solution (1 μl) was added to the bacterial cell suspension. After incubation for 10 min in the dark, cells were observed using fluorescent microscopy (Nikon eclipse Ti, Nikon Corporation, Japan). The images were digitally recorded, and using NIS-Elements AR (V.4.0), were adjusted for brightness and contrast.

## Results and Discussion

### Enzymatic Synthesis of BAVE and CAVE

**Fig. 1:**
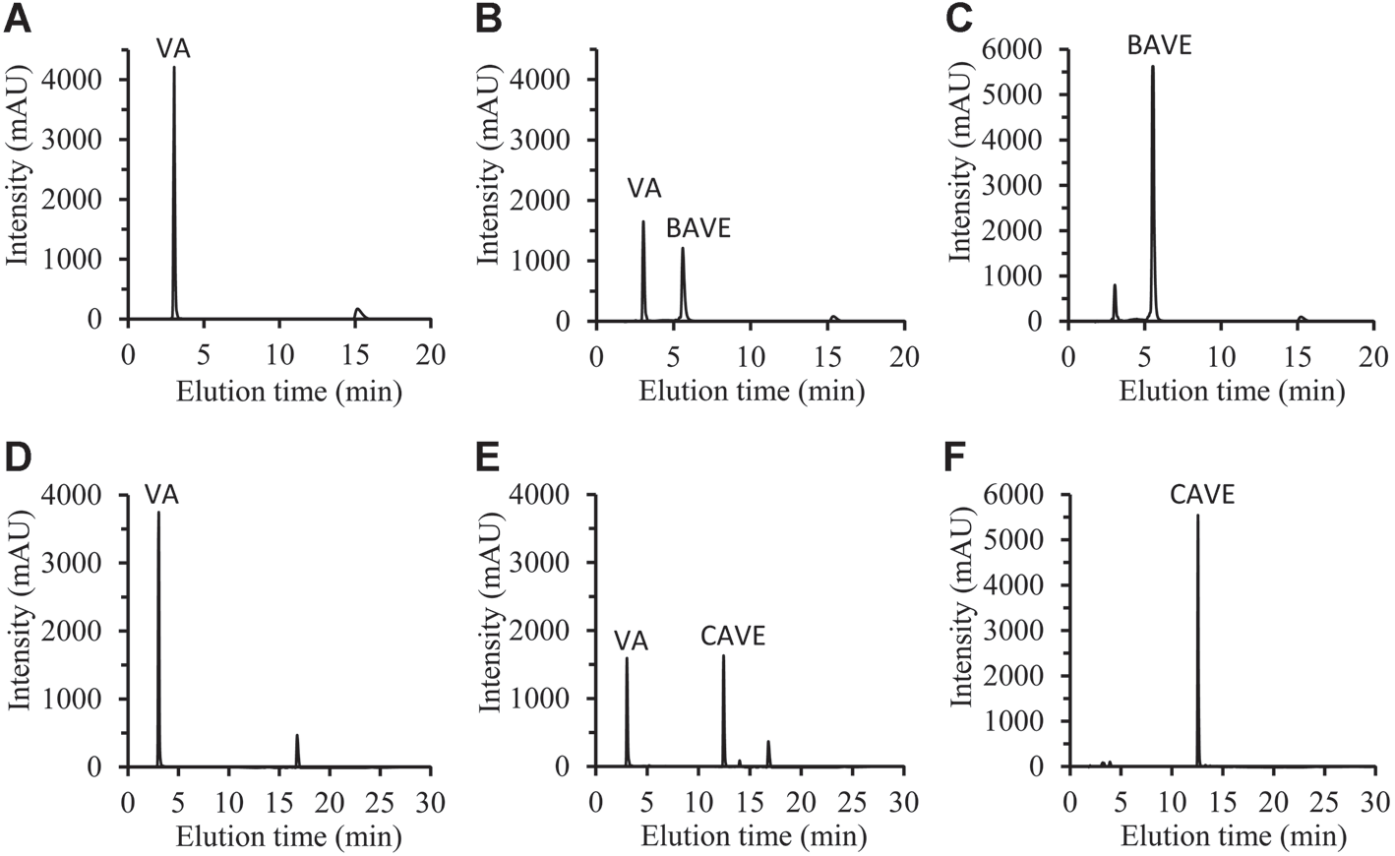
High-performance liquid chromatography chromatograms. (**A**) Reaction mixture containing VA and TBN was analyzed at 0 h. (**B**) Reaction mixture containing VA and TBN was analyzed at 1 h. (**C**) Purified BAVE was analyzed. (**D**) Reaction mixture containing VA and TCN was analyzed at 0 h. (**E**) Reaction mixture containing VA and TCN was analyzed at 0.5 h. (**F**) Purified CAVE was analyzed.

**Scheme 1:**
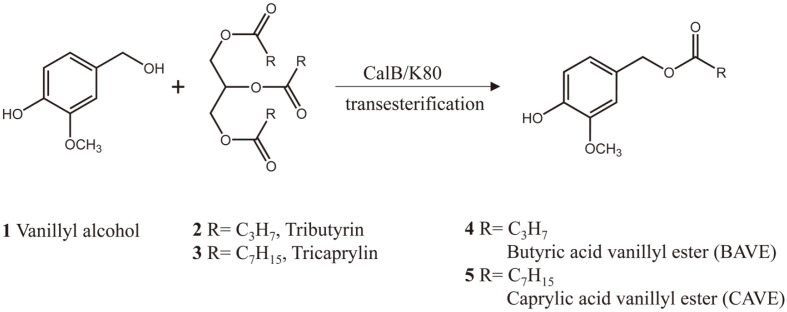
Lipase-mediated transesterification of vanillyl alcohol and triglycerides.

Lipase-mediated transesterification was performed for the production of BAVE and CAVE (Scheme 1). The main reaction parameters of the molar ratio of substrates, the amount of lipase used, and the reaction time were optimized to obtain large amounts of the target products. After the enzyme reaction, the amounts of VA, BAVE, and CAVE were analyzed through HPLC with C18 column. HPLC analysis showed that they were eluted in the order VA, BAVE, and CAVE at (3, 5.5, and 12.5) min, respectively (Fig. 1).

**Fig. 2:**
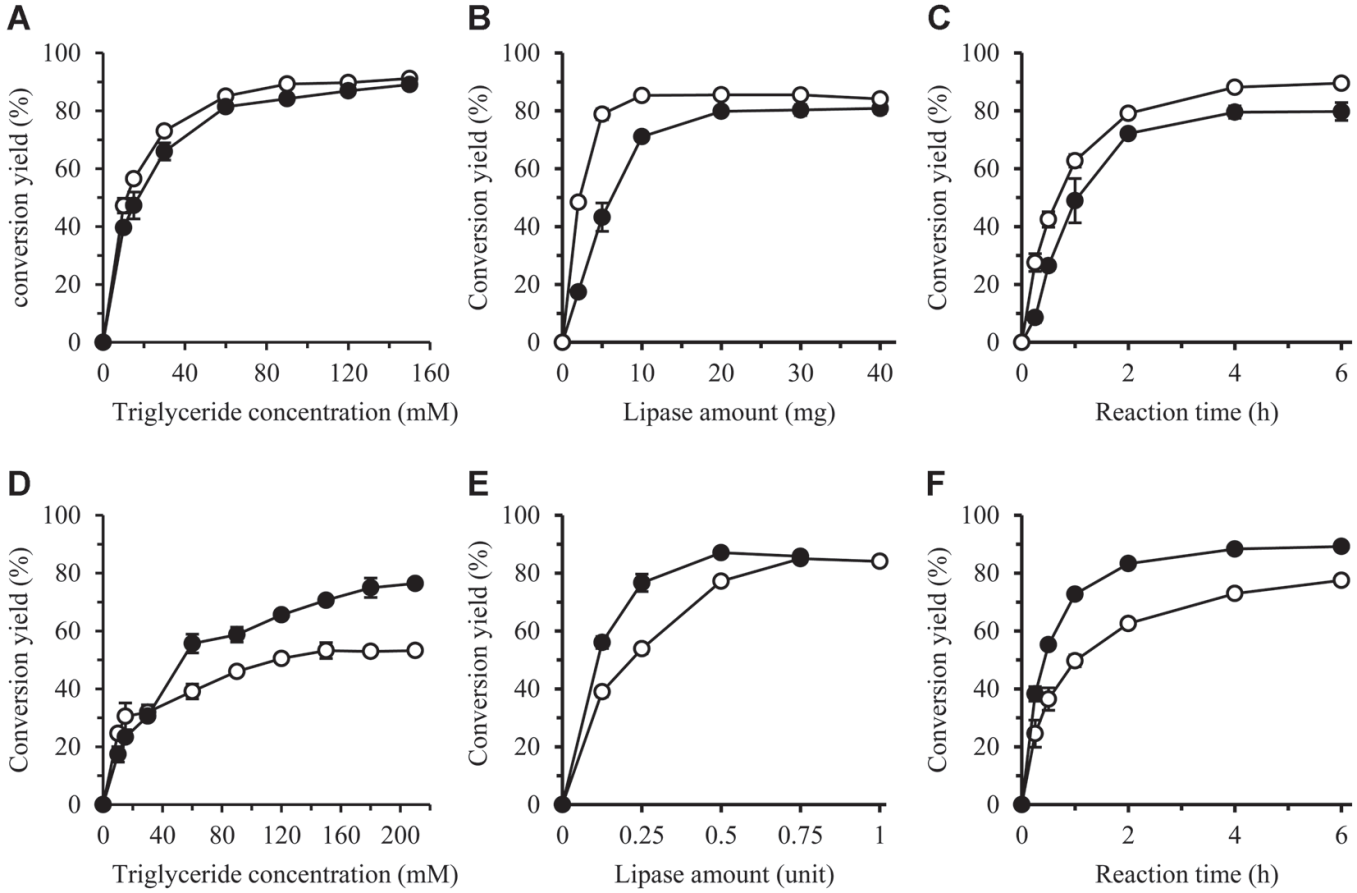
Optimization of transesterification reaction parameters using CalB (A-C) and K80 (D-F) lipases. The lipase activities of CalB and K80 were 2.7 and 10.7 U/g of bead, respectively. (**A**) and (**D**) The effects of the molar ratio of VA and triglyceride were investigated. The concentration of VA was fixed at 30 mM, while the concentration of triglyceride was changed. (**B**) and (**E**) The effect of lipase amount was investigated. (**C**) and (**F**) The time course of conversion yield was calculated under optimized conditions. ○, TBN; ●, TCN.

First, the reaction optimization was performed using CalB enzyme, which is the most widely used enzyme in industry. We used CalB enzyme immobilized to acrylic resins, because this enzyme reaction was performed in an organic solvent. The reaction of VA and oil was carried out by changing their molar ratio. As the molar ratio of oil to VA increased, conversion yield increased. The optimal molar ratio was determined as 3:9 (30 mM VA and 90 mM TBN) for BAVE, and 3:6 (30 mM VA and 60 mM TCN) for CAVE, and the conversion yields were (89 and 84) %, respectively (Fig. 2A).

Second, the amount of CalB lipase was optimized. In the case of BAVE and CAVE synthesis, conversion yield increased with increasing lipase amount, and the highest conversion yield was obtained when CalB was added by as much as (10 and 20) mg, respectively. The conversion yields were (85 and 79) %, respectively (Fig. 2B).

Finally, the reaction time was optimized. Conversion yield increased with reaction time course, and both products showed the highest conversion yield after 4 h. The conversion yields were measured as (88 and 79) % for BAVE and CAVE, respectively (Fig. 2C).

Next, the same reaction was performed using K80 lipase. This is an enzyme derived from *P. vulgaris*, and is a recombinant enzyme produced from *E. coli*. As the enzyme has high activity and organic solvent stability, it has been used in many transesterification reactions [20, 21]. In this research, we used K80 enzymes immobilized to MA-DVB resin.

The optimal molar ratio of BAVE synthesis was determined as 3:15 (VA 30 mM and TBN 150 mM), and that of CAVE synthesis was determined as 3:18 (VA 30 mM and TCN 180 mM). Conversion yields were measured as (53 and 79) %, respectively (Fig. 2D). Second, the optimum amounts of lipase were determined as 0.75 U for BAVE synthesis, and 0.5 U for CAVE synthesis, and the conversion yields were (85 and 87) %, respectively (Fig. 2E). Finally, the optimum reaction times were 6 h for BAVE synthesis, and 4 h for CAVE synthesis. The conversion yields were (77 and 89) %, respectively (Fig. 2F).

 The initial specific conversion rates of the CalB and K80 enzymes were calculated from the data in Figs. 1C and 1F. For CalB, the values for TBN and TCN were (1.11 and 0.62) min^-1^, respectively. This means that CalB has higher substrate selectivity for TBN than for TCN. For K80, the values for TBN and TCN were (0.91 and 1.61) min^-1^, respectively. This means that K80 has higher substrate selectivity for TCN than TBN. According to these results, CalB enzyme is suitable for BAVE production, while K80 enzyme is suitable for CAVE production.

### Purification and Structure Analysis of Products

After the reaction, the reaction product was purified from the reaction mixture through preparative LC, and concentrated by rotary evaporation method (Figs. 1C and 1F). The structure of the purified compound was analyzed by 300 MHz NMR (Figs. S1 and S2 of the Supplementary Information (SI)). The NMR results showed that the structure of the purified CAVE was the same structure as that of compound 5 in Scheme 1. The purified BAVE also had the same structure as that of compound 4 in Scheme 1, but it was found to contain a residual amount of VA (Fig. 1C).

### DPPH Radical Scavenging Activity Assay

**Fig. 3:**
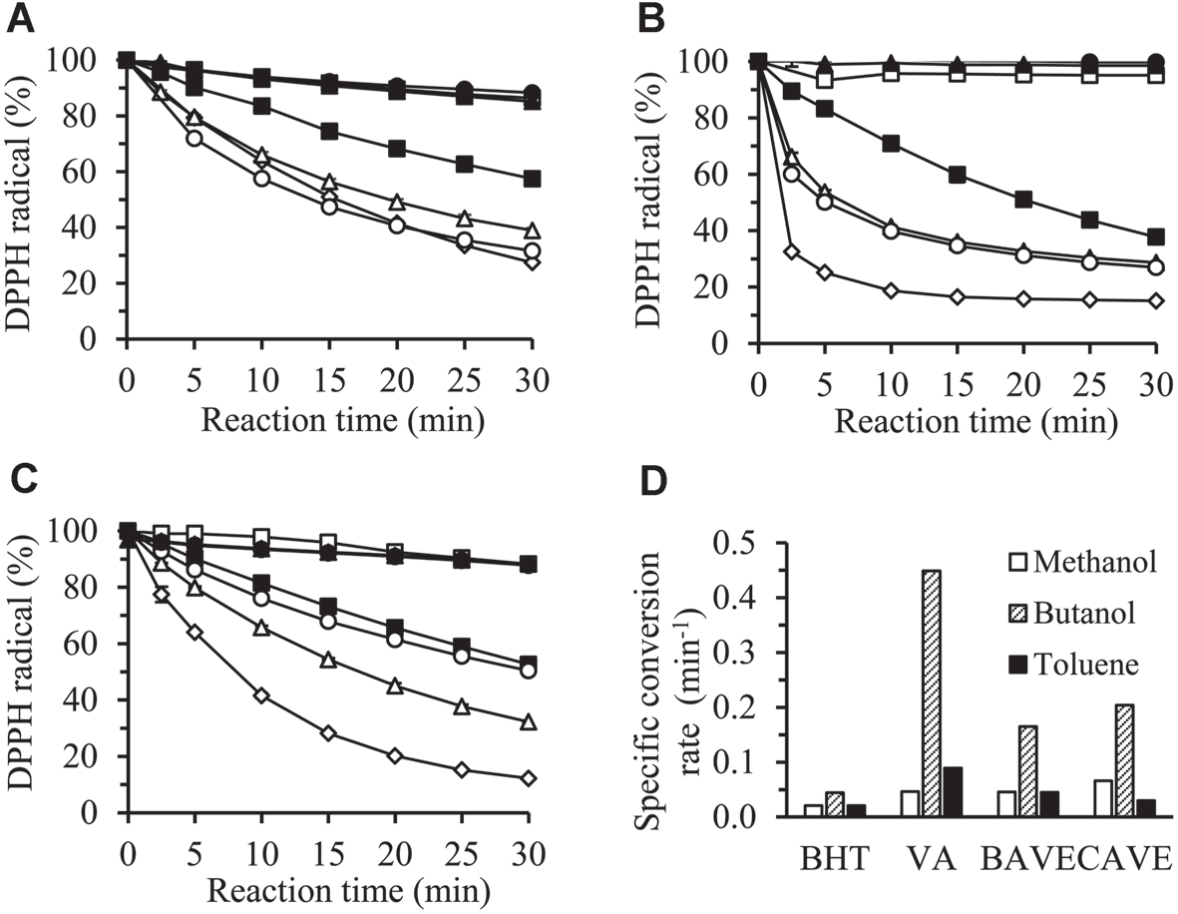
DPPH radical scavenging activities of BAVE and CAVE. Assay was performed in methanol (**A**), butanol (**B**), and toluene (**C**). □, solvent; ■, 1 mM BHT; ◇, 1 mM VA; △, 1 mM BAVE; ▲, 1 mM TBN; ○, 1 mM CAVE; ●, 1 mM TCN. (**D**) The specific conversion rate of each antioxidant in methanol, butanol, and toluene was calculated by the following equation: $k(t-t_{0})=-\ln\frac{\left[s_{t}\right]}{\left[s_{0}\right]},$ where [s_t_] is the residual DPPH concentration at a given time t, and [s_0_] is the DPPH concentration at 0 min.

The DPPH radical scavenging activities of BAVE and CAVE were tested in three organic solvents: methanol (log P -0.69), butanol (log P 0.16), and toluene (log P 2.68). After 30 min of reaction time, VA, BAVE, and CAVE showed high DPPH radical scavenging activity in all solvents (Fig. 3), which was higher than BHT used as positive control. TBN and TCN showed no DPPH radical scavenging activity in all solvents (Fig. 3). When methanol was used as a reaction solvent, BAVE and CAVE scavenged (61.1 and 68.4) % DPPH radicals, respectively (Fig. 3A). In the case of butanol, (71.3 and 73) % (Fig. 3B) were removed, respectively; and in toluene, (67.7 and 49.6) %(Fig. 3C) were removed.

The initial specific conversion rates of BHT, VA, BAVE, and CAVE were calculated in the three solvents (Fig. 3D). The initial conversion rates of VA, BAVE, and CAVE were significantly higher in assays using butanol, than in assays using methanol and toluene. This shows that the DPPH radical scavenging activity of these compounds is significantly changed by the logP value of the solvent. In addition, the conversion rates of VA, BAVE, and CAVE were rather higher than that of BHT, which means that these compounds have high antioxidant activity.

### CD and CT Assay

**Fig. 4:**
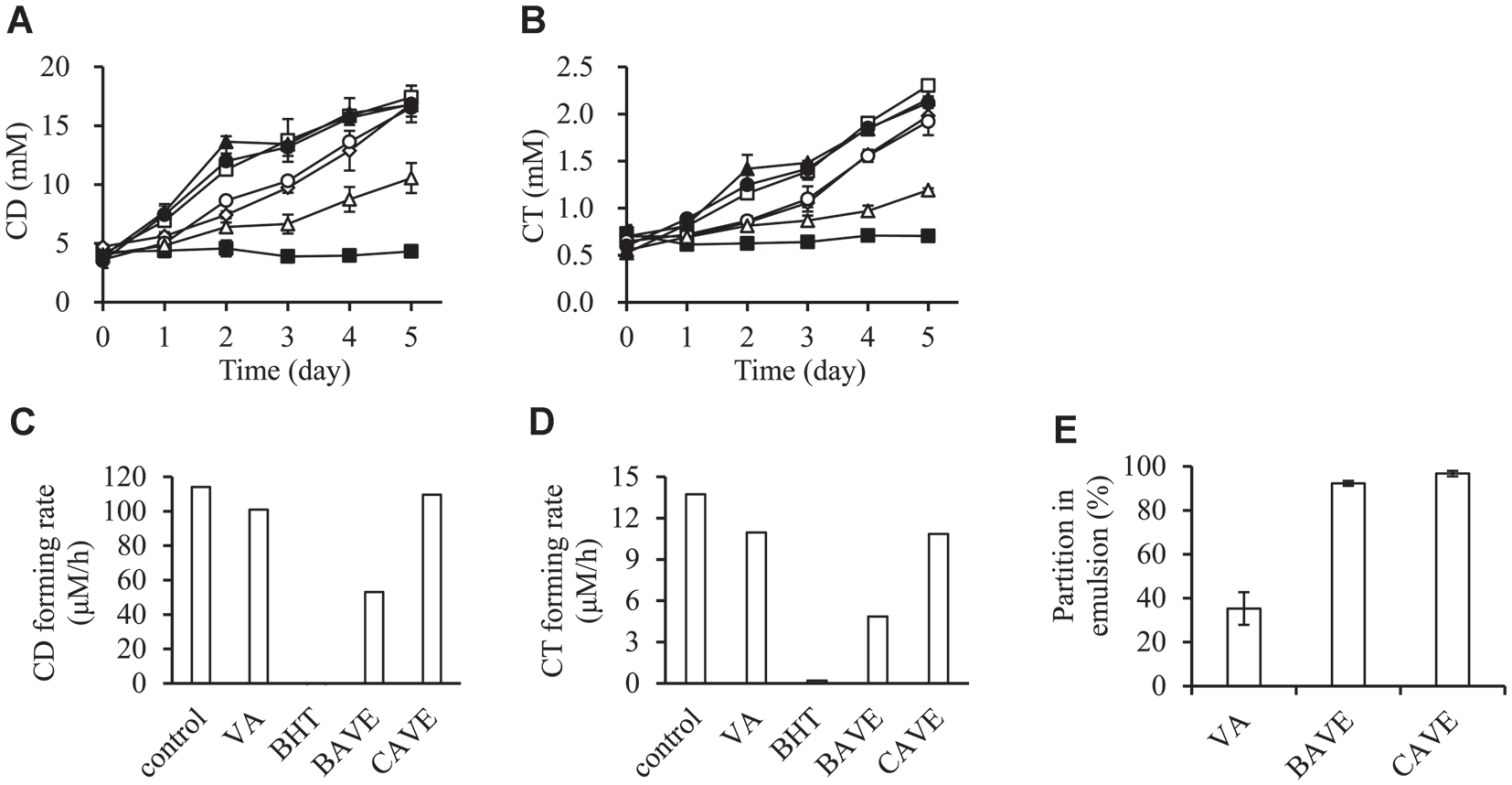
Effects of antioxidants on MO-in-water emulsion. MO-in-water emulsion was prepared with 10% MO, 1% Tween 20, and 1 mM test compounds. MO-in-water emulsion was stored at 35°C for 5 days. (**A**) Formation of CD was measured on the basis of absorbance at 237 nm. (**B**) Formation of CT was measured on the basis of absorbance at 270 nm. □, None; ■, 1 mM BHT; ◇, 1 mM VA; △, 1 mM BAVE; ▲, 1 mM TBN; ○, 1 mM CAVE; ●, 1 mM TCN. (**C**) and (**D**) CD or CT-forming rate was calculated from the linear equation obtained by the least-square method. (**E**) Partition behavior of VA, BAVE, and CAVE in MO-in-water emulsion system. Emulsion was prepared with 50% MO, 1% Tween 20, and 1 mM test compounds. After 24 h, emulsion and aqueous phases were analyzed using HPLC.

To investigate the applicability of BAVE and CAVE as antioxidants in food, we measured how BAVE and CAVE inhibited the production of CD and CT in lipid oxidation process in emulsion. BHT, a positive control, completely inhibited the production of CD and CT during the storage period of 5 days; however, TBN and TCN did not inhibit the production of CD and CT at all. The CD production in the VA-emulsion was similar to that of the negative control (no additive) on the 5th day. The CT production in VA-emulsion was 86%, compared to the negative control at day 5. BAVE showed higher antioxidant activity than VA; CD production was 60.6% of the negative control on the 5th day (Fig. 4A), CT production was 51.8% (Fig. 4B), compared to the negative control. However, when CAVE was used, CD and CT production were similar to those obtained by VA treatment (Figs. 4A and 4B). In addition, the CD-forming rate and CT-forming rate calculated from the linear equation obtained by least-square method also showed the same results as those measured after the 5-day reaction (Figs. 4C and 4D).

Interestingly, BAVE has more potently prevented CD and CT production than VA and CAVE. To investigate the reasons for the different antioxidant activity in emulsion, we measured how much VA, BAVE, and CAVE were distributed in aqueous phase and emulsion phase (including oil and interface phase). In the case of VA, only 35.3%was distributed in the emulsion phase (Fig. 4E). In contrast, BAVE and CAVE were distributed in emulsion phase by as much as (92.3 and 96.7) %, respectively (Fig. 4E). Therefore, VA was not able to effectively prevent CD production in emulsion, because of the low solubility for emulsion phase.

Oxidation in emulsion is known to occur at the interface of oil and water. According to a previous report, the degree of lipid oxidation and the distribution of antioxidants in the interface are inversely related [37]. The hydrophilic–lipophilic balance (HLB) values of VA, BAVE, CAVE, Tween 20, and MO are (18.0, 14.8, 11.9, 16.7, and 12), respectively. CAVE that has similar HLB value of MO will be distributed at the inner part of emulsion, and BAVE with medium HLB values will be distributed at the edge of emulsion. This may be explained as BAVE having a high inhibitory effect on CD and CT production, because the lipid oxidation process progresses rapidly on the interface of oil and water.

### Determination of MIC

Microdilution assay was used to investigate the inhibitory activity of BAVE and CAVE in bacterial growth. The culture experiments were conducted on four bacteria (*A. faecalis*, *B. coagulans*, *B. subtilis*, and *P. fluorescens*), and MIC was calculated at a concentration that inhibited the growth of bacteria by 90%. As a result of the experiment, CAVE showed antibacterial activity for all four bacteria. MIC for Gram-positive bacteria, *B. coagulans* and *B. subtilis*, was (15.0 and 57.4) μM, respectively; and MIC for Gram-negative bacteria, *A. faecalis* and *P. fluorescens*, was (991 and 200) μM, respectively (Table 1). As such, Gram-positive bacteria were more sensitive to CAVE than were Gram-negative bacteria. However, the reaction substrates and BAVE did not have any antibacterial activity up to 1 mM for *A. faecalis*, and 2 mM for the other three bacteria.

According to the previous literature, the mechanism of the antibacterial activity of amphipathic molecules, such as fatty acids and monoglycerides, is as follows: (1) increased membrane permeability and cell lysis, (2) disruption of electron transport chain, (3) uncoupling oxidative phosphorylation, and (4) inhibition of membrane enzymatic activities and nutrients uptake [38]. Among them, CAVE is assumed to act on the cell membrane of bacteria, thereby increasing permeability, resulting in cell lysis. In the next section, we perform additional experiments to prove this assumption.

According to another report in the literature, essential oil showed more potent antibacterial activity against gram positive bacteria than gram negative bacteria. In other words, Gram-negative bacteria have more complex and harder outer membranes, and lipopolysaccharide present in this membrane inhibits the diffusion of hydrophobic molecules. However, Gram-positive bacteria did not have an outer membrane, and the peptidoglycan cell wall was less resistant to hydrophobic compounds [39]. In our experiments, CAVE showed also more potent antimicrobial activity against Gram-positive bacteria, than did Gram-negative bacteria.

### Zeta Potential Measurements

**Fig. 5:**
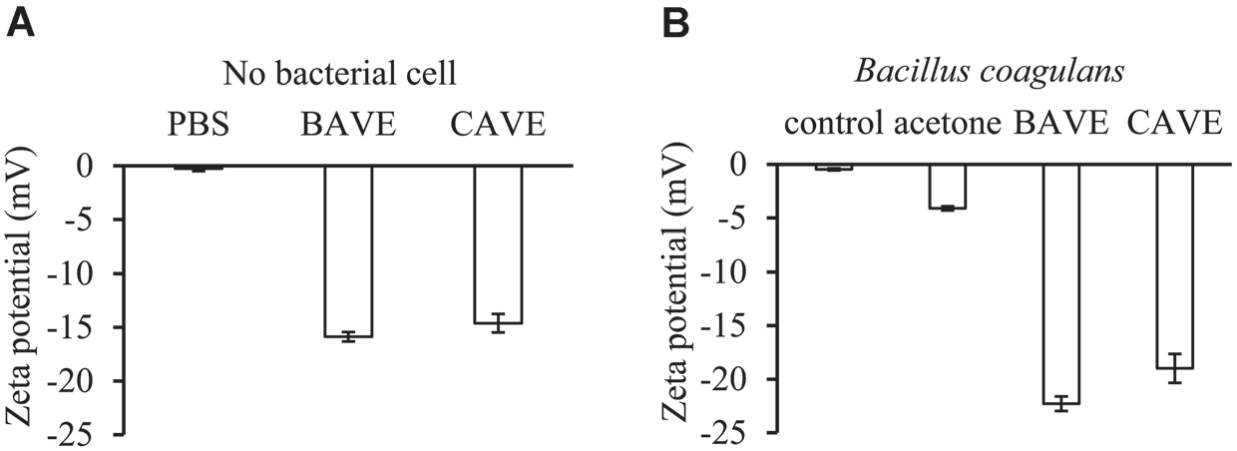
Zeta potential measurements. (**A**) Zeta potential was measured for BAVE and CAVE. (**B**) Zeta potential was measured for *B. coagulans* after incubation for 4 h in PBS, 5% acetone, 2 mM BAVE, and 2 mM CAVE.

The zeta potential was measured to determine whether BAVE and CAVE were inserted into the *B. coagulans* cell membrane. The phenolic compound is known to have a negative charge by hydroxyl groups connected to the aromatic ring [21, 40]. BAVE and CAVE in PBS showed negative charge of (-16 and -15) mV, respectively (Fig. 5A). In addition, *B. coagulans* treated with BAVE and CAVE increased negative charge from (-5 to (-22 and -18)) mV, respectively (Fig. 5B). These results indicate that both BAVE and CAVE were inserted into the cell membrane of *B. coagulans*.

### PI Uptake Assay and Fluorescent Microscopy

**Fig. 6:**
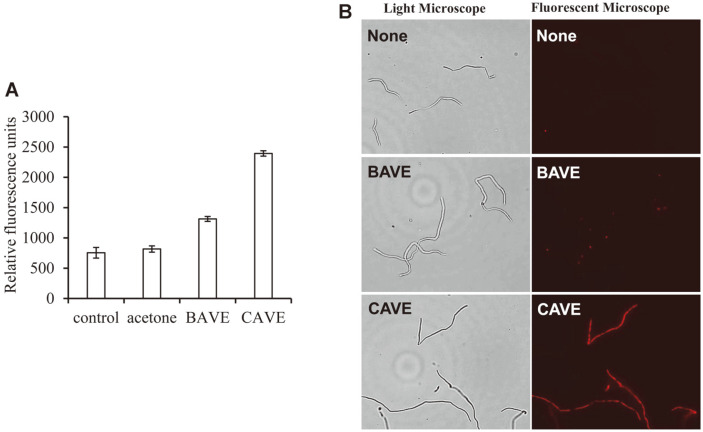
PI uptake assay and fluorescent microscopy. (**A**) After treating BAVE and CAVE for 4 h, the levels of PI uptake of *B. coagulans* were measured. (**B**) *B. coagulans* treated with PBS (none), BAVE, and CAVE were stained with PI, and observed with light microscopy and fluorescent microscopy.

**Scheme 2:**
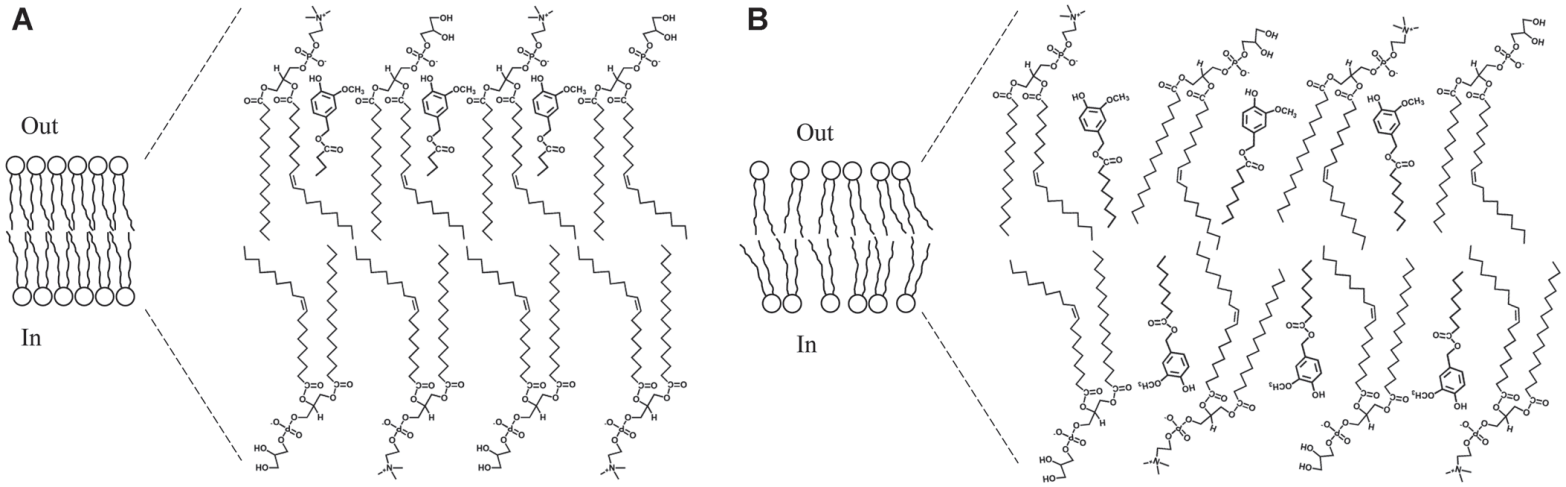
A proposal for the structural change of cell membrane by BAVE or CAVE. (**A**) Insertion of BAVE into *B. coagulans* cytoplasmic membrane consisting of phosphatidyl glycerol and phosphatidyl ethanolamine. (**B**) Insertion of CAVE into *B. coagulans* cytoplasmic membrane and destabilization of the membrane structure.

The PI uptake assay using fluorescence spectrophotometry was performed to check whether CAVE changed the permeability of the cell membrane of *B. coagulans*. PI is a compound that is used as an indicator of membrane integrity, because if the structure of the cell membrane is destroyed, it can pass through the cell membrane. In other words, PI is known to have red fluorescence by dyeing DNA and RNA after entering the dead cells, or the ones with reversibly damaged membranes [41]. CAVE treatment with *B. coagulans* showed 3.17 times more fluorescence than that without CAVE treatment (Fig. 6A). This means that PI has passed through the cell membrane, and entered the cell. However, BAVE, which did not show antimicrobial activity, also showed 1.74 times more fluorescence than control (Fig. 6A). This means that BAVE can also bind to the cell membrane, and partially affect the structure of the cell membrane. To investigate this visually, PI dyeing was performed, and then observed by fluorescent microscopy (Fig. 6B). In the case of BAVE treatment, only a part of the bacteria showed weak fluorescence, but most of the bacteria did not show fluorescence. However, when CAVE was treated, almost all bacteria showed strong fluorescence. These results indicate that CAVE destroyed the cell membrane stability of *B. coagulans* and increased the permeability of materials, leading to the death of bacteria. On the other hand, BAVE was inserted into the cell membrane, but the length of the chain was short, so the effect on the permeability of the cell membrane was weak, which was assumed to not be fatal to the bacteria (Scheme 2).

Taken together, BAVE and CAVE were synthesized by transesterification using lipase. These compounds had similar antioxidant activity to VA. In particular, BAVE showed high activity to prevent lipid oxidation in the emulsion system. CAVE containing a medium chain fatty acid also had antibacterial activity. In particular, it showed strong activity for the Gram-positive bacteria. It seemed to destroy the structural stability of the cell membrane, and kill bacteria. As such, CAVE has both antioxidant and antibacterial activities, so it is expected that it will be used as a bi-functional material for the food and cosmetics industries.

## Supplemental Materials



Supplementary data for this paper are available on-line only at http://jmb.or.kr.

## Figures and Tables

**Table 1:** Antibacterial activities of various compounds.

Bacterial strain	MIC (μM)				

	VA	TBN	TCN	BAVE	CAVE
*Alcaligenes faecalis* KCTC2678	>1000	>1000	>1000	>1000	991
*Bacillus coagulans* KCCM11715	>2000	>2000	>2000	>2000	15.0
*Bacillus subtilis* KCTC2189	>2000	>2000	>2000	>2000	57.4
*Pseudomonas fluorescens* KCCM41443	>2000	>2000	>2000	>2000	200

*MIC (minimum inhibitory concentration) was calculated as a concentration that causes 90% growth inhibition compared to control bacteria.
