# Supplementary material for: Antioxidant and Antibacterial Activity of Caprylic Acid Vanillyl Ester Produced by Lipase-Mediated Transesterification
Source: J Microbiol Biotechnol. 2020 Nov 10;31(2):317–26. doi: 10.4014/jmb.2010.10018 (PMC9723275; doi:10.4014/jmb.2010.10018)
Supplement: Supplementary file 1 [file jmb-31-2-317-supple.pdf]

J. Microbiol. Biotechnol.
https://doi.org/10.4014/jmb.2010.10018

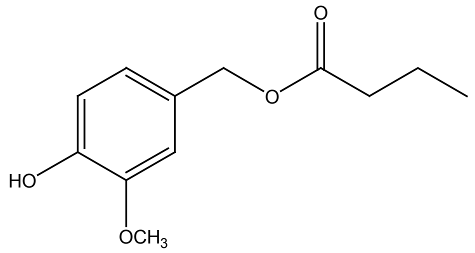

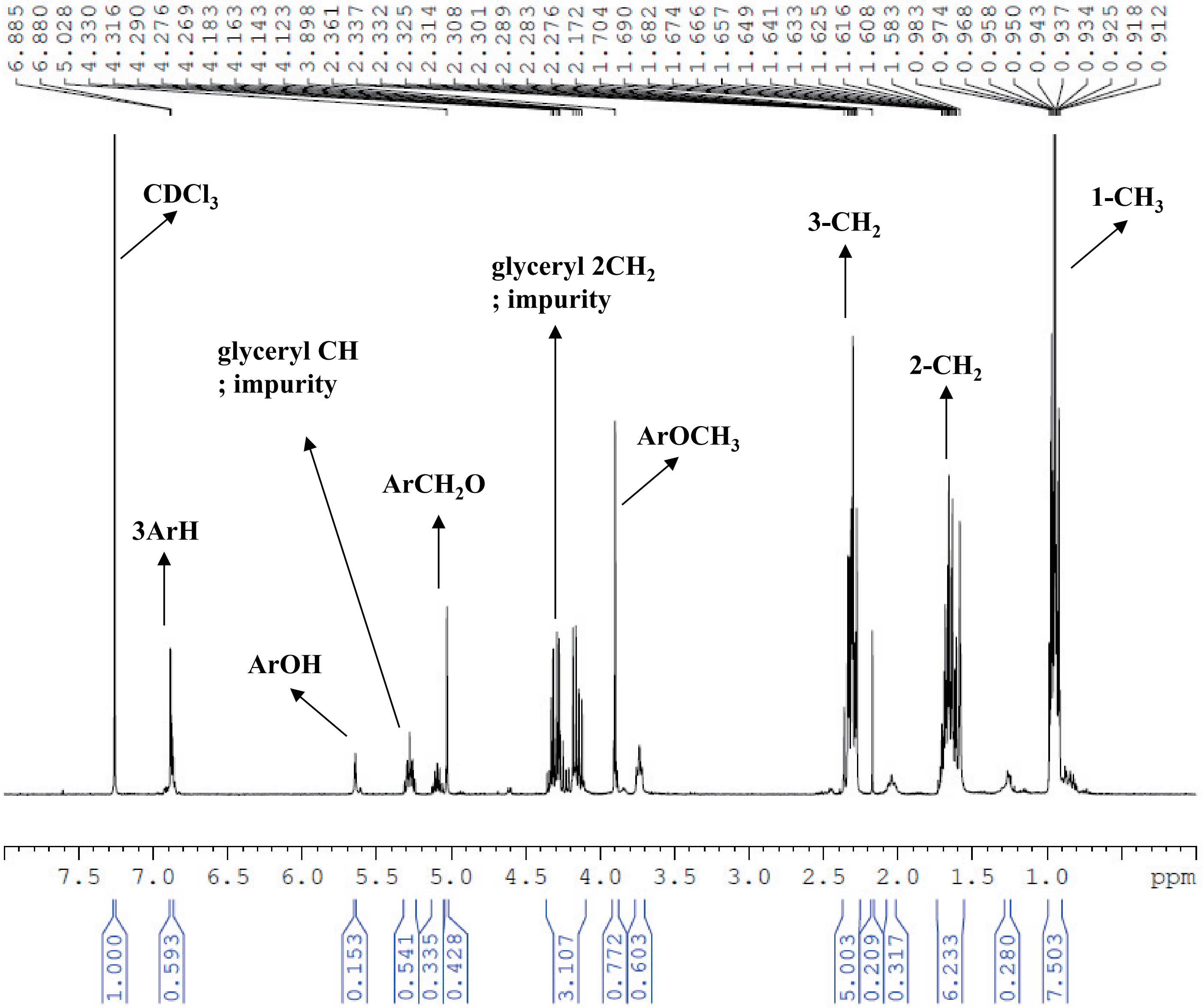

Supplementary Fig. S1. ^{1}H-NMR ($CDCl_3$) spectrum of BAVE

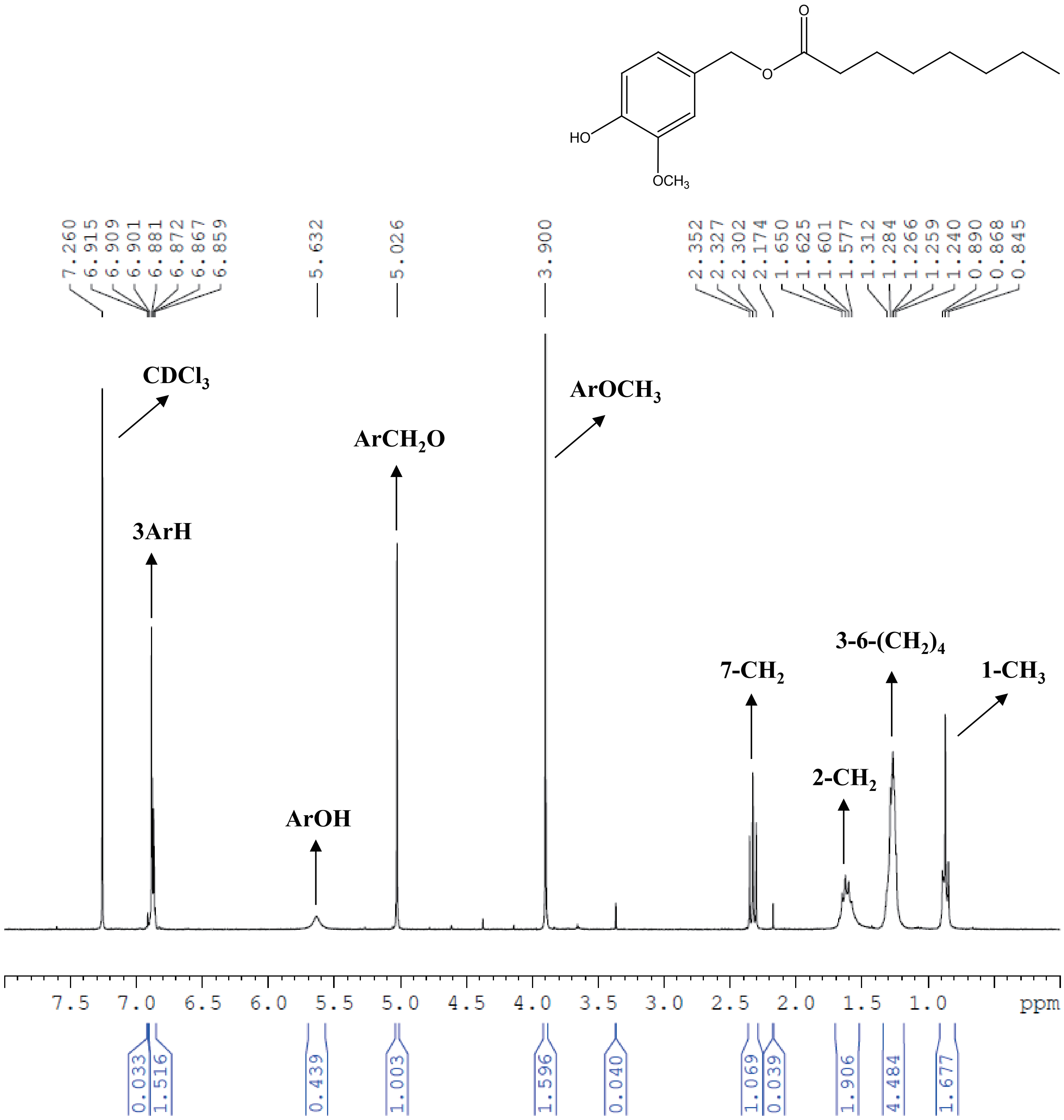

Supplementary Fig. S2. ^{1}H-NMR ($CDCl_3$) spectrum of CAVE
